# School Nursing Practice and Factors Associated With Job Satisfaction Among School Nurses Supporting Children With Medical Complexity in Japanese Elementary Schools

**DOI:** 10.1111/jjns.70083

**Published:** 2026-10-13

**Authors:** Mutsumi Seki, Rie Okamoto, Shizuko Omote, Satomi Ikeuchi, Yuko Noguchi, Yutaro Takahashi

**Affiliations:** ^1^ Division of Health Science Graduate School of Medical Sciences, Kanazawa University Ishikawa Japan; ^2^ Department of Nursing Faculty of Nursing, Niigata College of Nursing Niigata Japan; ^3^ Faculty of Health Sciences Institute of Medical, Pharmaceutical and Health Sciences, Kanazawa University Ishikawa Japan; ^4^ Kanazawa University Ishikawa Japan; ^5^ Department of Public Health Nursing Graduate School of Medicine, Mie University Tsu Japan

**Keywords:** children with medical complexity, job satisfaction, nurse–teacher collaboration, school nurses, working conditions

## Abstract

**Aim:**

The number of children with medical complexity attending mainstream elementary schools in Japan has increased, highlighting the critical role of school nurses in supporting their care. We aimed to clarify the current status of school nursing practice and to explore factors associated with job satisfaction among school nurses supporting children with medical complexity in Japan.

**Methods:**

We conducted an exploratory and descriptive cross‐sectional web‐based survey among school nurses working in elementary schools across nine prefectures in Japan with a high proportion of children having medical complexity (estimate of eligible nurses = 768; valid responses = 137). We measured job satisfaction levels using a visual analog scale and subsequently performed multiple regression analysis.

**Results:**

Most school nurses were employed in non‐regular positions (93.7%); 65.6% worked as the sole nurse at their school. Advanced medical procedures were more frequently performed in schools with multiple school nurses. Job satisfaction was positively associated with collaborative achievement (*β* = 0.539, *p* < 0.001) and consultations with school staff (*β* = 0.214, *p* = 0.004) and was negatively associated with developing individualized care manuals (*β* = −0.184, *p* = 0.013).

**Conclusions:**

This study suggests that effective interprofessional collaboration, appropriate staffing, and supportive working environments may be important for sustaining the school nursing workforce caring for children with medical complexity. These findings provide empirical evidence for considering working conditions and organizational support in developing a sustainable school nursing system in Japan.

## 1 Introduction

Children with medical complexity (CMC), often involving multi‐organ conditions and dependence on medical technology, require continuous care (Cohen et al. 2011), and their numbers are increasing worldwide (Nkoy et al. 2019). Ensuring equitable access to education for these children is a critical global issue. The World Health Organization and United Nations Educational, Scientific and Cultural Organization (2021) recommend adequate school nurse staffing and the establishment of training programs to support safe school attendance for students with complex health needs.

School health systems vary substantially across countries. In the United States, the National Association of School Nurses (2021) advocates that every school should be staffed with a registered nurse, and school nursing has been institutionalized as a specialized profession requiring formal certification (Lineberry and Ickes 2015). The American Academy of Pediatrics has issued a policy statement promoting the placement of a full‐time registered nurse in every school (Council on School Health 2016). Nevertheless, regional disparities in employment settings and reports of moral distress among school nurses have been noted (Willgerodt et al. 2024), highlighting the need for sustainable school health systems that are capable of responding to increasingly complex student health needs. Previous international studies have also indicated that organizational support and interprofessional collaboration influence nurses' job satisfaction and retention (Lu et al. 2012; San Martín‐Rodríguez et al. 2005).

In Japan, children who require ongoing medical care in daily and social life are legally defined as children requiring medical care (Ministry of Health, Labour and Welfare 2021). These children often require medical procedures such as suctioning, gastrostomy care, intermittent catheterization, and oxygen therapy, as well as coordinated support across health care and educational settings. In this study, this population is described as CMC. In Japan, every school employs a Yogo teacher—a licensed educator responsible for health monitoring, first aid, and health education; however, this role does not require nursing qualifications. Nurses are assigned to a school only when CMC attend the school and require medical support. Consequently, providing individualized medical care for CMC in all school settings poses important challenges. Recent legal and policy developments in Japan, including ratification of the Convention on the Rights of Persons with Disabilities in 2014 (Ministry of Foreign Affairs of Japan 2014) and enactment of the Act on Support for Children Requiring Medical Care and Their Families in 2021 (Ministry of Health, Labour and Welfare 2021), have strengthened the legal framework for inclusive education. As a result, the number of CMC attending mainstream schools (i.e., schools other than specialized schools) has increased, the need for medical care in school settings has grown, and the placement of nurses in mainstream schools has risen. Despite the growing demand for medical care in school settings, challenges have been reported, including situations in which parents are required to accompany their children to school and provide care owing to the absence of a school nurse (Ministry of Education, Culture, Sports, Science, and Technology 2022a). Similar patterns have been observed internationally, where families of CMC often assume primary responsibility for care owing to gaps in health care and school‐based support systems (Kuo et al. 2012; American Academy of Pediatrics 2014). These challenges underscore the need to better understand the working conditions of school nurses who support CMC.

Previous international research has highlighted the importance of collaboration and working conditions in sustaining the nursing workforce (Aiken et al. 2002; Lu et al. 2012). Recent studies from the United States have also highlighted challenges related to the school nurse workforce, including workforce shortages and staffing issues, as well as the expanding roles and responsibilities of school nurses (Willgerodt et al. 2024). In Japan, studies on nurses working in mainstream schools have focused primarily on their roles (Shimizu and Katsuda 2015) and collaboration with teachers. Previous international studies of hospital nurses have suggested that psychological stability and collaborative relationships are associated with nurses' job satisfaction (Aiken et al. 2002). However, these findings may not be applicable to mainstream school settings because the professional roles and working environments of school nurses differ substantially from those of hospital nurses. Previous research on school nurses has identified supervisory support, workplace climate, and professional autonomy as important factors associated with job satisfaction (Foley et al. 2004). However, to the best of our knowledge, no large‐scale surveys have yet comprehensively examined the working conditions, care practices, and job satisfaction of school nurses in elementary schools following the recent legal reforms.

Given these gaps, in this exploratory and descriptive study, we aimed to clarify the current status of school nursing practice, working conditions, collaboration, and job satisfaction among school nurses supporting CMC in elementary schools of Japan and to explore factors associated with job satisfaction. Addressing these issues is important for improving the working environment of school nurses and supporting the provision of high‐quality nursing care for CMC in school settings. The findings of this study provide empirical evidence on school nursing practice and factors associated with job satisfaction among school nurses supporting CMC.

## 2 Methods

### 2.1 Study Design

This study followed an exploratory and descriptive cross‐sectional design using a web‐based survey.

### 2.2 Definition of Key Terms

Under the Japanese Act on Support for Children Requiring Medical Care and Their Families, children requiring medical care are defined as those for whom ongoing medical care is essential to maintain daily life and social functioning (Ministry of Health, Labour and Welfare 2021). Examples of such care include suctioning, gastrostomy care, intermittent catheterization, and oxygen therapy. These children often have complex health care needs and require coordinated support across health care and educational settings. To facilitate international understanding, children requiring medical care in Japan are referred to as CMC in this study.

School nurses were defined as licensed nurses assigned to schools to provide health care support and nursing care for CMC.

Yogo teachers were defined as licensed educators responsible for school health services in Japan who are not required to hold nursing qualifications.

### 2.3 Participants and Sampling Procedure

According to the 2021 national survey on medical care in schools conducted by the Ministry of Education, Culture, Sports, Science and Technology (2022a), the number of elementary school students requiring medical care and the number of nurses assigned to elementary schools were reported for each prefecture. However, the survey did not identify individual schools enrolling children who required medical care or schools that employed nurses. Therefore, it was not possible to directly identify eligible schools or nurses. To efficiently reach nurses supporting children who require medical care in elementary schools, prefectures were selected based on the proportion of elementary schools that enrolled children requiring medical care. Using data from the School Basic Survey (Ministry of Education, Culture, Sports, Science, and Technology 2022b) and the 2021 national survey on medical care in schools (Ministry of Education, Culture, Sports, Science, and Technology 2022a), we identified nine prefectures in which the proportion of elementary schools enrolling children who required medical care was 8% or higher. According to the national survey, these nine prefectures included 5037 elementary schools and 768 nurses were assigned to elementary schools. Then, using publicly available information from prefectural boards of education including school names and locations, 4864 elementary schools were confirmed and included as survey sites. Based on these data, the estimated number of eligible nurses in this study was 768.

### 2.4 Procedure

Because individual nurses could not be directly identified, a school‐based recruitment approach was used. Research cooperation requests were sent to the principals of all elementary schools (4864 schools) in the nine prefectures. The principals of schools with assigned nurses were asked to distribute the study invitation to school nurses; the invitation included a QR code linking to the web‐based survey. The method of distributing the study materials to school nurses within each school was left to the discretion of the principal. Electronic informed consent was obtained from all participants before they began the questionnaire. Participation was voluntary, and responses were collected anonymously. Questionnaires containing complete data for the primary outcome and variables required for the main analyses were considered valid responses. The survey was conducted online from March to April 2024. This study was approved by the Ethics Committee of the authors' affiliated institution (Approval No. 023‐09, approved on October 30, 2023) and another institutional review board (Approval No. 111132‐1, approved on December 22, 2023).

### 2.5 Instrumentation

The collected data comprised age, professional qualifications, employment status, years of experience working in elementary schools, years of clinical experience, presence or absence of pediatric nursing experience, number of nurses, and number of CMC in the respondent's school. Furthermore, use of the *Training Program for Nurses Beginning Work in Schools* (hereafter referred to as the “training program”) (Mizuho Information and Research Institute Inc. 2021) and the *Manual for the Implementation of Medical Care in Schools* (hereafter referred to as the “manual”) (Japan Home‐Visit Nursing Foundation 2019) was assessed using a 4‐point scale. Perceptions regarding CMC, specifically the degree of emotional attachment to CMC and the perceived difficulty in assessing the health status and medical care needs of CMC, were evaluated using a 4‐point scale. Job satisfaction and salary satisfaction were assessed using a visual analog scale (0–10). Because the purpose of this exploratory and descriptive study was to obtain an overall assessment of satisfaction and to capture subtle variations as continuous variables, single‐item visual analog scale measures were used. Additionally, no validated instrument specifically designed for school nurses working in mainstream elementary schools in Japan was identified. School nursing practice, including the implementation of medical care and the scope of roles and responsibilities, was evaluated with reference to the *Report on Securing Training Opportunities* (Japan Home‐Visit Nursing Foundation 2021). Collaboration within schools, encompassing the extent of collaboration with teachers and Yogo teachers, was assessed using a 4‐point scale. To evaluate the sense of collaborative achievement in the school setting, the Nurse–Teacher Collaboration Scale (NTCS), developed for medical care providers, was applied (Kanayama and Iwai 2014). This scale is based on the theoretical framework proposed by San Martín‐Rodríguez et al. (2005) and comprises three subscales: organizational/system factors (13 items), professional interpersonal factors (7 items), and daily interpersonal factors (8 items). Each item is rated on a 4‐point scale, yielding a total score ranging from 28 to 112. The reliability and validity of the scale have been confirmed (Kanayama and Iwai 2014). Although the NTCS was originally developed and validated in Japan, it has also been applied in a recent longitudinal study examining burnout–collaboration patterns among medical care workers in special needs schools (Kanayama et al. 2016).

### 2.6 Data Analysis

Data were collected anonymously using SurveyMonkey (SurveyMonkey, San Mateo, CA, USA), and statistical analyses were performed using IBM SPSS version 27 (IBM Corp., Armonk, NY, USA). Descriptive statistics were used to summarize participant characteristics, perceptions regarding CMC, collaboration with school staff, and nursing practices. Cases with missing data for variables included in each analysis were excluded. For example, analyses comparing nurse staffing patterns were conducted among participants with available data for this variable. In examining the associations with job satisfaction, after confirming normality, Pearson's correlation coefficients and *t*‐tests were used to examine the relationships between job satisfaction and attributes, working conditions, experience, extent of attachment, role and responsibility, salary satisfaction, extent of collaboration with teachers and Yogo teachers, and NTCS scores. Variables originally assessed using 4‐point scales were dichotomized for analysis. Categories with similar conceptual meanings were combined to ensure sufficient numbers in each category and to facilitate stable statistical analysis. We assessed the degree of attachment using a 4‐point scale and recoded this into high (always or generally) and low (rarely or not at all). Similarly, collaboration with teachers and Yogo teachers was assessed with a 4‐point scale and recoded into two groups: established (well or generally established) and not established (rarely or not established). Perceived difficulty in clinical judgment, assessed using a 4‐point scale, was recoded into difficult (difficult or sometimes difficult) and not difficult (rarely difficult or not difficult). In the univariate analyses, variables with *p* < 0.10 were considered for inclusion in the multivariable model to avoid excluding potentially relevant predictors. To explore factors associated with job satisfaction, stepwise multiple linear regression analysis was performed. This exploratory approach was adopted to identify potential factors associated with job satisfaction. In the multivariable analyses, a significance level of *p* < 0.05 was used. Variables were excluded when substantial imbalances were observed between categories of dummy variable (Wissmann et al. 2014). Multicollinearity was assessed using the variance inflation factor.

Initial translation and language editing were performed using ChatGPT (OpenAI). All outputs were reviewed and verified by the authors, who take full responsibility for the content of the manuscript.

## 3 Results

Of 768 eligible nurses, 181 responded to the survey (response rate: 23.6%), of whom 137 provided valid responses and were included in the analysis (75.7% of respondents).

### 3.1 Participant Characteristics

Participants' mean age was 48.2 years (range: 24–76), and 119 (86.9%) were in non‐regular employment. The mean length of employment was 2.2 years (standard deviation [SD] = 1.9). Among respondents, 84 (61.3%) had no prior pediatric nursing experience, and 86 (62.8%) were in single‐nurse placement. Eight respondents (5.8%) reported that they had used a training program, and 22 (16.1%) reported that they had used a manual. A total of 130 participants (94.9%) reported having an emotional attachment to CMC, and 107 (78.1%) reported difficulties with assessing changes in the status of CMC or making clinical judgments related to their medical care. The mean job satisfaction score was 6.6 (SD = 2.2) on a 10‐point scale, and the mean salary satisfaction score was 4.4 (SD = 2.7) on a 10‐point scale (Table 1). Scores for job and salary satisfaction were approximately normally distributed, with no ceiling or floor effects observed.

**TABLE 1:** Participants' characteristics (*N* = 137).

Item	*n* (%)	*M* (SD)
Basic characteristics		
Age (years)		48.2 (10.6)
Professional qualification		
Registered nurse (including public health nurse, midwife)	118 (86.1)	
Licensed practical nurse	6 (4.4)	
No response	13 (9.5)	
Employment status		
Non‐regular employment	119 (86.9)	
Regular employment	8 (5.8)	
No response	10 (7.3)	
Service in elementary schools (years)		2.2 (1.9)
Less than 1	29 (21.2)	
1 to < 2	32 (23.4)	
2 to < 3	25 (18.2)	
3 to < 4	20 (14.6)	
4 to < 5	13 (9.5)	
5 or more	18 (13.1)	
Clinical experience (years)		15.1 (12.5)
Pediatric nursing experience		
Yes	53 (38.7)	
No	84 (61.3)	
Number of nurses assigned per school day		
1	86 (62.8)	
2 or more	45 (32.8)	
No response	6 (4.4)	
Number of CMC cared for per day		
1	112 (81.8)	
2 or more	20 (14.6)	
No response	5 (3.6)	
Participation in training program		
Has participated in a training program	8 (5.8)	
Has seen a training program	18 (13.1)	
Knows about a training program but has not participated	9 (6.6)	
Does not know about any training program	102 (74.5)	
Use of manual		
Has used a manual	22 (16.1)	
Has seen a manual	37 (27.0)	
Knows about a manual but has not seen one	12 (8.8)	
Does not know about any manual	66 (48.2)	
Perceptions regarding CMC		
Attachment to CMC		
Always feels attached	98 (71.5)	
Generally feels attached	32 (23.4)	
Rarely feels attached	5 (3.6)	
Does not feel attached	1 (0.7)	
No response	1 (0.7)	
Perceived difficulty in assessing CMC health status and care needs		
Feels it is difficult	42 (30.7)	
Sometimes feels it is difficult	65 (47.4)	
Rarely feels it is difficult	25 (18.2)	
Does not feel it is difficult	5 (3.6)	
Job satisfaction		
Job satisfaction (0–10 scale)		6.6 (2.2)
Salary satisfaction (0–10 scale)		4.4 (2.7)
No response	2 (1.5)	

*Note:* Percentages are calculated based on the total sample (*N* = 137). “No response” indicates missing data. Training program refers to training for nurses beginning work in schools. Manual is for the implementation of medical care in schools.

Abbreviations: CMC, children with medical complexity; *M*, mean; SD, standard deviation.

### 3.2 Implementation of Medical Care According to Nurse Staffing Pattern

The three medical care procedures most frequently implemented in schools were intermittent catheterization (33.6%), gastrostomy/enterostomy care (30.5%), and tracheostomy cannula suctioning (29.0%). In multiple‐nurse placements, the implementation rates of suctioning, oxygen therapy, mechanical ventilation, nasogastric tube feeding, and airway clearance devices were significantly higher than the rates in single‐nurse placements (Table 2). Nurses in schools with two or more nurses were significantly more likely to report perceived difficulty in medical judgment than those in single‐nurse placements (*p* = 0.028).

**TABLE 2:** Implementation of medical care and comparison according to nurse staffing pattern (*N* = 131).

Type of medical care	*n* (%)	Nurse staffing pattern		*p*
		1 nurse (*N* = 86)	≥ 2 nurses (*N* = 45)	
Intermittent catheterization				
Yes	44 (33.6)	29	15	0.964^a^
No	87 (66.4)	57	30	
Gastrostomy/enterostomy				
Yes	40 (30.5)	22	18	0.089^a^
No	91 (69.5)	64	27	
Suctioning at tracheostomy site (cannula)				
Yes	38 (29.0)	18	20	0.005^a^
No	93 (71.0)	68	25	
Oral/nasal suctioning				
Yes	30 (22.9)	11	19	< 0.001^a^
No	101 (77.1)	75	26	
Oxygen therapy				
Yes	28 (21.4)	12	16	0.004^a^
No	103 (78.6)	74	29	
Blood glucose monitoring/insulin administration				
Yes	25 (19.1)	20	5	0.093^a^
No	106 (80.9)	66	40	
Mechanical ventilation				
Yes	18 (13.7)	4	14	< 0.001^a^
No	113 (86.3)	82	31	
Nasogastric tube feeding				
Yes	16 (12.2)	6	10	0.011^a^
No	121 (87.8)	80	35	
Assisted expectoration				
Yes	12 (9.2)	4	8	0.018^b^
No	119 (90.8)	82	37	
Medication administration				
Yes	10 (7.6)	4	6	0.079^b^
No	121 (92.4)	82	39	
Ostomy/urinary diversion				
Yes	4 (3.1)	2	2	0.426^b^
No	127 (96.9)	84	43	
Central venous nutrition				
Yes	3 (2.3)	0	3	NA
No	128 (97.7)	86	42	
Indwelling bladder catheter				
Yes	2 (1.5)	1	1	0.571^b^
No	129 (98.5)	85	44	
Enema				
Yes	2 (1.5)	2	0	NA
No	129 (98.5)	84	45	

*Note:* *N* = 131 represents participants with available data on nurse staffing pattern. Participants with missing responses for this variable were excluded from the analysis.

Abbreviation: NA, not applicable.

^a^ *χ* ^2^ test.

^b^ Fisher's exact test.

### 3.3 Implementation of Nursing Roles and Responsibilities

The primary roles of school nurses in our survey included responding to emergencies (73.0%), managing medical equipment and supplies (65.7%), involvement in consultations regarding medical care of CMC with teachers and school staff (58.4%), and involvement in consultations with parents regarding medical care for CMC (58.4%) (Table 3).

**TABLE 3:** Nurses' roles and responsibilities (*N* = 137).

Item	*n* (%)
Responding to emergencies	100 (73.0)
Management of medical devices and supplies	90 (65.7)
Responding to consultation requests from teachers and school staff regarding medical care for CMC	80 (58.4)
Responding to consultation requests from parents regarding medical care for CMC	80 (58.4)
Supporting the education of students without medical care needs	67 (48.9)
Developing individualized care manuals for CMC	55 (40.1)
Providing advice to parents on medical care	54 (39.4)
Preparing emergency manuals	53 (38.7)
Communicating and reporting to physicians and other medical professionals	45 (32.8)
Promoting understanding of medical care needs among school staff	31 (22.6)
Promoting social participation of students with CMC	31 (22.6)
Coordinating with external organizations (medical, health, welfare, others)	18 (13.1)
Providing care while CMC commutes to and from school	9 (6.6)

*Note:* The reported roles include direct medical care as well as consultation, coordination, educational support, and communication with school staff, parents, and health care professionals.

Abbreviation: CMC, children with medical complexity.

### 3.4 Status of Collaboration and Sense of Achievement

Among respondents, 114 (83.2%) reported that collaboration with teachers was either established or generally established; 90 participants (65.7%) reported that collaboration with Yogo teachers was established or generally established. The mean total score on the NTCS, representing the sense of collaborative achievement, was 70.9 (SD = 10.6).

### 3.5 Factors Related to Job Satisfaction

In the univariate analysis, job satisfaction was significantly associated with attachment to CMC (*p* < 0.001), salary satisfaction (*r* = 0.299, *p* < 0.001), development of individualized care manuals for CMC based on a physician's orders (*p* = 0.057), involvement in consultations with teachers and school staff (*p* = 0.017), and the NTCS total score (*r* = 0.570, *p* < 0.001) (Table 4). No significant associations were found with the basic characteristics.

**TABLE 4:** Univariate associations with job satisfaction among nurses (*N* = 137).

Item	*N* (%)	*M* (SD)		*r*	*p*
Perceptions					
Attachment to CMC					
Present	130 (94.9)	6.7 (2.1)			< 0.001^a^, ^b^
Limited	6 (4.4)	2.7 (1.8)			
No response	1 (0.7)				
Job satisfaction					
Salary satisfaction	135 (98.5)	4.4 (2.7)		0.299	< 0.001^c^
No response	2 (1.5)				
Roles and responsibilities					
Developing individualized care manuals for CMC					
Yes	55 (40.1)	6.1 (2.1)			0.057^a^
No	82 (59.9)	6.8 (2.2)			
Responding to consultation requests from teachers and school staff regarding medical care for CMC					
Yes	80 (58.4)	6.9 (2.0)			0.017^a^
No	57 (41.6)	6.0 (2.4)			
Collaboration and cooperation					
Status of collaboration with teachers					
Collaboration group	114 (83.2)	6.9 (2.0)			< 0.001^a^
Non‐collaboration group	23 (16.8)	4.8 (2.5)			
Status of collaboration with Yogo teachers					
Collaboration group	90 (65.7)	6.9 (1.9)			0.034^d^
Non‐collaboration group	47 (34.3)	5.9 (2.6)			
NTCS total score	137	70.9 (10.6)		0.570	< 0.001^c^

*Note:* Only variables meeting the screening criterion (*p* < 0.10) are presented. Attachment was recoded into high (always or generally) and low (rarely or not at all). Collaboration with teachers and Yogo teachers was recoded into established (well or generally established) and not established (rarely or not established).

Abbreviations: CMC, children with medical complexity; *M*, mean; *N*, number of participants; NTCS, Nurse–Teacher Collaboration Scale; SD, standard deviation.

^a^ *t*‐test.

^b^ Variables with highly imbalanced category frequencies were excluded from multiple regression analysis.

^c^ Pearson's correlation coefficient.

^d^ Welch test.

In multiple regression analysis, the NTCS total score (*β* = 0.539, *p* < 0.001) and involvement in consultations with teachers and school staff (*β* = 0.214, *p* = 0.004) were significantly and positively associated with job satisfaction. Involvement in the development of individualized care manuals for CMC based on physician's orders was significantly and negatively associated with job satisfaction (*β* = −0.184, *p* = 0.013). The adjusted *R* ^2^ of the model was 0.365 (Table 5). Attachment to CMC was not included in the multiple regression model because of the highly imbalanced distribution of responses.

**TABLE 5:** Factors associated with job satisfaction among nurses (multiple regression analysis).

Independent variables	*B*	SE	*β*	95% CI	*p*
NTCS total score	0.11	0.01	0.539	0.08–0.14	< 0.001
Responding to consultation requests from teachers and school staff regarding medical care for CMC (Yes/No)	0.96	0.33	0.214	0.32–1.60	0.004
Involvement in developing individualized care manuals for CMC (Yes/No)	−0.83	0.33	−0.184	−1.47 to −0.18	0.013

*Note:* Yes = 1, No = 0 (dummy variables). Involvement in medical care consultations refers to participation in consultations with teachers and school staff regarding medical care needs for CMC. Model statistics: *R* ^2^ = 0.379; adjusted *R* ^2^ = 0.365; *N* = 135; Variance inflation factor range = 1.02–1.11.

Abbreviations: *β*, standardized regression coefficient; *B*, unstandardized regression coefficient; CI, confidence interval; CMC, children with medical complexity; NTCS, Nurse–Teacher Collaboration Scale; SE, standard error.

## 4 Discussion

### 4.1 Interpretation of Key Findings

This study is among the few multi‐prefecture surveys in Japan to examine both the current status of school nursing practice for CMC and factors associated with job satisfaction among school nurses working in Japanese elementary schools. The present findings provide empirical insights into school nursing practice in Japan and factors associated with job satisfaction among school nurses supporting CMC.

### 4.2 Characteristics of School Nurses

Previous reports have noted that many school nurses transition from other nursing fields after gaining substantial clinical experience and that opportunities for career advancement within school nursing are limited (McCabe et al. 2024). Our findings of an older age distribution, short tenure as a school nurse, and a high proportion of non‐regular employment may also suggest that school nursing represents a career transition pathway for nurses with prior clinical experience. However, non‐regular employment conditions may also limit long‐term retention and pose challenges to maintaining a stable school nursing workforce. Given the limited and specialized nature of school nursing, supportive working environments that enable school nurses to exercise their professional expertise and continue practicing school nursing may be important for sustaining this workforce. Many school nurses reported working as the sole nurse in their school and therefore assumed primary responsibility for providing medical care to CMC. This concentration of responsibility may place substantial professional demands on individual nurses, underscoring the need for organizational support and collaborative working environments. Another notable finding was that nearly all participating school nurses reported becoming emotionally attached to CMC. This appears to be an important characteristic of the study population. This finding is also consistent with previous qualitative research suggesting that school nurses develop attachment to children through ongoing relationships, derive personal satisfaction from supporting children's growth and development, and may regard this attachment as one of the reasons for continuing to work in school nursing (Kruger et al. 2009).

The short tenure and high proportion of non‐regular employment observed among Japanese school nurses differed from the employment structure of school nursing in the United States. Although variations exist across states, school nursing in the United States is relatively institutionalized in terms of the qualification requirements and scope of practice (National Association of School Nurses 2021; Willgerodt et al. 2024). However, concerns related to low income and part‐time employment among school nurses in the United States have also been reported (Willgerodt et al. 2024), suggesting that employment instability may represent a broader structural issue within school nursing systems. In both contexts, improving working conditions appears to be essential for sustaining the school nursing workforce.

### 4.3 Nursing Practice in Supporting CMC

According to respondents, school nursing practice primarily involved assistance with bladder management and feeding, including intermittent catheterization and gastrostomy care, thereby directly supporting daily school participation among CMC. High‐risk procedures, including suctioning and mechanical ventilation, were more frequently implemented in schools with multiple‐nurse placements, which may reflect staffing arrangements in response to the greater medical complexity of CMC requiring high‐risk care. Similarly, the greater perceived difficulty in medical judgment among nurses in multiple‐nurse placements may also reflect the greater medical complexity of CMC supported by these nurses. Given the developmental and clinical complexity of caring for CMC, the lack of pediatric nursing experience among many school nurses represents an important challenge.

Despite the availability of training programs and manuals, their reported use was limited. At the time of the survey, training programs were mainly provided in the form of educational materials for self‐learning, and opportunities for school nurses to participate in structured training programs may have been limited. Previous studies have also reported insufficient training opportunities for school nurses; this context may partly explain the limited use of training programs observed in the present study. Participation in a training program was not associated with job satisfaction. This may indicate that the training programs available at the time of the survey were not sufficiently accessible or comprehensive to influence the work experiences of school nurses. This interpretation is consistent with previous research indicating that insufficient organizational support and limited professional development opportunities may contribute to nurses' intention to leave their positions and to reduced job satisfaction (Flinkman et al. 2010).

Regarding staffing arrangements, although single‐nurse placement has often been considered structurally vulnerable, nurses in schools with multiple‐nurse staffing were more likely to report perceived difficulty in medical judgment. This finding may reflect that schools supporting children with greater medical complexity are more likely to have multiple‐nurse staffing arrangements. Thus, perceived clinical difficulty in medical judgment may be more influenced by the complexity of medical conditions among CMC than by staffing numbers alone. These findings highlight the importance of providing organizational support tailored to the complexity of CMC's medical needs rather than relying solely on staffing numbers.

### 4.4 Factors Influencing Job Satisfaction

The strongest factor associated with job satisfaction in this study was a sense of collaborative achievement. This finding suggests that school nurses experience greater job satisfaction when interprofessional collaboration functions effectively across organizational/systemic, interprofessional, and interpersonal domains, and when nurses perceive that this collaboration has achieved meaningful outcomes. Thus, perceiving collaboration as effective and successful may be more important than merely participating in collaborative activities. This interpretation is also supported by previous research demonstrating that effective interprofessional collaboration, characterized by mutual role understanding and shared goals, contributes to professional satisfaction among health care providers (San Martín‐Rodríguez et al. 2005). Additionally, collaboration is reportedly associated with burnout over time in longitudinal research using the NTCS (Kanayama et al. 2016).

Although both collaborative achievement and responding to consultation requests are related to collaboration, they reflect different aspects of collaborative functioning. Collaborative achievement represents an overall perception of effective collaboration, whereas responding to consultation requests reflects opportunities for school nurses to exercise and demonstrate their professional expertise within collaborative practice. Such interactions may enhance job satisfaction by enabling school nurses to experience professional recognition and role clarity as health care professionals within schools. This interpretation is consistent with previous research indicating that professional recognition and role clarity contribute to job satisfaction among nurses (Lu et al. 2012). Previous research among school nurses has also identified supervisory support, workplace climate, and professional autonomy as important predictors of job satisfaction (Foley et al. 2004).

Involvement in developing individualized care manuals for CMC based on physicians' orders was negatively associated with job satisfaction. Preparing such manuals is an essential responsibility to ensure safe care; however, this task requires considerable coordination with physicians and may also reflect involvement in medically complex cases. Because many school nurses work as the sole nurse in their school, a school nurse who develops an individualized care manual is also responsible for implementing and managing the medical care based on that manual. Consequently, this concentrated responsibility may partly explain the negative association between involvement in manual development and job satisfaction. Given that most school nurses in our survey were employed in non‐regular positions with limited working hours, this responsibility represents a substantial burden. These findings highlight the need for organizational support systems to distribute the responsibility for developing individualized care manuals rather than placing this responsibility on individual school nurses.

Japanese school nurses in our study reported lower levels of satisfaction with their salary than with the work itself. This finding may reflect professional and cultural values emphasizing commitment to patient care, as suggested in previous studies of nursing professionalism (Pang et al. 2003). However, this explanation alone does not fully account for the discrepancy between salary satisfaction and job satisfaction observed in the present study. School nursing may represent a career transition pathway for nurses with prior clinical experience, permitting continued employment despite relatively low financial rewards. Structural improvements in employment conditions and institutional support may therefore contribute not only to job satisfaction but also to workforce sustainability while ensuring continuous support for CMC.

### 4.5 Implications

The findings of this study give rise to several important implications for school health systems supporting CMC. From a policy perspective, employment systems and staffing standards should be better aligned with the professional responsibilities of school nurses. Establishing more stable employment frameworks, clarifying staffing standards, and providing organizational support for high‐responsibility tasks—such as developing individualized care manuals for CMC—are critical steps toward building a sustainable school nursing system.

From a practice perspective, schools should foster collaborative environments in which school nurses can effectively apply their professional expertise. Promoting mutual understanding of roles and professional expertise between school nurses and school staff, together with shared goals and effective communication, may enhance a sense of collaborative achievement and improve job satisfaction. These efforts may be particularly important for school nurses working in single‐nurse placements, where opportunities for consultation and collaborative problem‐solving are limited.

From a workforce sustainability perspective, differences in school nurse staffing arrangements and nurses' clinical experience may affect not only the continuity and quality of care for CMC but also the long‐term sustainability of the school nursing workforce. Ensuring the continuous availability of school nurses through stable support systems is essential for providing continuous, high‐quality care for CMC. Standardizing training systems for school nurses across municipalities, while also promoting continuing professional development and workforce recruitment, may help to ensure maintenance of the school nursing workforce and contribute to continuous, high‐quality support for CMC. Overall, the present findings highlight the importance of strengthening workforce sustainability and collaborative practices within school health systems to ensure that CMC receive continuous, high‐quality care and support.

### 4.6 Limitations and Future Directions

The strengths of this study include its survey design covering multiple Japanese prefectures, as well as the use of validated measures to assess collaborative achievement. Nevertheless, several study limitations should be considered. First, the relatively low response rate may limit generalizability of the findings. Second, the cross‐sectional design precludes causal inference regarding the relationship between working conditions and job satisfaction. Third, participants were recruited through school principals, which may have introduced selection bias. Because the researchers could not contact school nurses directly, we were unable to confirm whether all eligible school nurses received the invitation or survey link. Therefore, school nurses with greater interest in the study or greater job satisfaction may have been more likely to participate. Additionally, because the analyses were exploratory in nature, the findings should be interpreted with caution and confirmed in future studies. Despite these limitations, the present study provides important insights into school nursing practice and job satisfaction among school nurses supporting CMC in Japan. Future research using longitudinal designs and more diverse recruitment strategies is needed to further clarify these relationships.

## 5 Conclusions

In this study, we identified several characteristics of the current school nursing workforce and factors associated with job satisfaction among school nurses supporting CMC in Japanese elementary schools. The findings revealed short average tenure, limited use of existing training resources, and a high prevalence of non‐regular employment. Job satisfaction was strongly associated with a sense of collaborative achievement and engagement in professional consultations, underscoring the importance of organizational support, professional recognition, and team cohesion. Strengthening organizational support, standardizing training systems, and enhancing collaborative school environments may be important for supporting sustainable school nursing systems. The present findings may contribute to the development of sustainable school nursing systems that provide continuous, high‐quality support for CMC.

## Author Contributions

Mutsumi Seki contributed to conceptualization, methodology, investigation, data curation, formal analysis, funding acquisition, project administration, and writing – original draft. Rie Okamoto and Shizuko Omote contributed to the methodology, supervision, and writing – review and editing. Satomi Ikeuchi, Yuko Noguchi, and Yutaro Takahashi contributed to the methodology and writing – review and editing. All authors approved the final version of the manuscript.

## Funding

This work was supported by the Japan Society for the Promotion of Science (22K10925).

## Ethics Statement

This study was conducted in accordance with the ethical principles of the Declaration of Helsinki (2013 revision). Approval was obtained from the Ethics Committee of Niigata College of Nursing (Approval No. 023‐09, approved on October 30, 2023) and the Medical Ethics Review Committee of Kanazawa University (Approval No. 111132‐1, approved on December 22, 2023). Permission to use the Nurse–Teacher Collaboration Scale was obtained from its developer. School principals and all participants were provided with written explanations describing the purpose of the study, research procedures, the voluntary nature of participation, and assurances of anonymity and confidentiality. All responses were collected anonymously, and no personally identifiable information was obtained. Electronic informed consent was obtained from all participants before they began the questionnaire.

## Conflicts of Interest

The authors declare no conflicts of interest.

## Supporting information


**Table S1:** Results of univariate analyses for all examined variables associated with job satisfaction (*N* = 137).

## Data Availability

The data that support the findings of this study are available from the corresponding author upon reasonable request.
