# Supplementary material for: School Nursing Practice and Factors Associated With Job Satisfaction Among School Nurses Supporting Children With Medical Complexity in Japanese Elementary Schools
Source: Jpn J Nurs Sci. 2026 Oct 13;23(4):e70083. doi: 10.1111/jjns.70083 (PMC13661291; doi:10.1111/jjns.70083)
Supplement: Supplementary file 1 [file JJNS-23-e70083-s001.docx]

| Supplementary Table S1. Results of univariate analyses for all examined variables associated with job satisfaction (N＝137) | | | | | | | | |
| --- | --- | --- | --- | --- | --- | --- | --- | --- |
| Item | |  | N | (%) | M | (SD) | r | p |
| **Basic characteristics** | |  |  |  |  |  |  |  |
| Nurses assigned per school day (n = 131) | |  |  |  |  |  |  |  |
|  | 1 |  | 86 | (65.6) | 6.5 | (2.4) |  | .813^†^ |
|  | 2 or more |  | 45 | (34.4) | 6.4 | (1.9) |  |  |
| CMC cared for per day (n = 132) | |  |  |  |  |  |  |  |
|  | 1 |  | 112 | (84.8) | 6.6 | (2.2) |  | .532^†^ |
|  | 2 or more |  | 20 | (15.2) | 6.3 | (2.0) |  |  |
| Service in elementary schools (years) | |  |  |  | 2.2 | (1.9) | 0.105 | .224^§^ |
| Clinical experience (years) | |  |  |  | 15.1 | (12.5) | −0.008 | .930^§^ |
| **Pediatric nursing experience** | |  |  |  |  |  |  |  |
|  | Yes |  | 53 | (38.7) | 6.5 | (2.2) |  | .321^‡^ |
|  | No |  | 84 | (61.3) | 6.6 | (2.2) |  |  |
|  |  |  |  |  |  |  |  |  |
| **Perceptions** | |  |  |  |  |  |  |  |
| Attachment to CMC | |  |  |  |  |  |  |  |
|  | Present |  | 130 | (94.9) | 6.7 | (2.1) |  | < .001^‡^ |
|  | Limited |  | 6 | (4.4) | 2.7 | (1.8) |  |  |
| Perceived difficulty in assessing CMC health status and care needs | |  |  |  |  |  |  |  |
|  | Difficult |  | 107 | (78.1) | 6.6 | (2.0) |  | .558^†^ |
|  | Not difficult |  | 30 | (21.9) | 6.3 | (2.7) |  |  |
| **Job satisfaction** | |  |  |  |  |  |  |  |
| Salary satisfaction | |  | 135 |  | 4.4 | (2.7) | 0.299 | < .001^§^ |
|  |  |  |  |  |  |  |  |  |
| **Roles and responsibilities** | |  |  |  |  |  |  |  |
| Developing individualized care manuals for CMC | |  |  |  |  |  |  |  |
|  | Yes |  | 55 | (40.1) | 6.1 | (2.1) |  | .057^‡^ |
|  | No |  | 82 | (59.9) | 6.8 | (2.2) |  |  |
| Responding to consultation requests from teachers and school staff regarding medical care for CMC | | | | | | | | |
|  | Yes |  | 80 | (58.4) | 6.9 | (2.0) |  | .017^‡^ |
|  | No |  | 57 | (41.6) | 6.0 | (2.4) |  |  |
| Provision of medical care | |  |  |  |  |  |  |  |
|  | Yes |  | 128 | (93.4) | 6.5 | (2.2) |  | .430^‡^ |
|  | No |  | 9 | (6.6) | 7.1 | (2.0) |  |  |
| Health management of CMC | |  |  |  |  |  |  |  |
|  | Yes |  | 114 | (83.2) | 6.5 | (2.2) |  | .509^‡^ |
|  | No |  | 23 | (16.8) | 6.8 | (2.1) |  |  |
| Documentation, management, and reporting of medical care | |  |  |  |  |  |  |  |
|  | Yes |  | 114 | (83.2) | 6.5 | (2.2) |  | .282^‡^ |
|  | No |  | 23 | (16.8) | 7.0 | (2.4) |  |  |
| Provision of medical care during off-campus school activities | |  |  |  |  |  |  |  |
|  | Yes |  | 103 | (75.2) | 6.6 | (2.2) |  | .391^‡^ |
|  | No |  | 34 | (24.8) | 6.3 | (2.2) |  |  |
| Assessment of CMC | |  |  |  |  |  |  |  |
|  | Yes |  | 93 | (67.9) | 6.4 | (2.1) |  | .138^‡^ |
|  | No |  | 44 | (32.1) | 7.0 | (2.4) |  |  |
| Recording of near-miss incidents and implementation of preventive measures | | | | | | | | |
|  | Yes |  | 29 | (21.2) | 6.1 | (2.2) |  | .262^‡^ |
|  | No |  | 108 | (78.8) | 6.7 | (2.2) |  |  |
| Guidance and advice for certified personnel performing designated medical procedures | | | | | | | | |
|  | Yes |  | 6 | (4.4) | 7.0 | (1.1) |  | .364^†^ |
|  | No |  | 131 | (95.6) | 6.5 | (2.2) |  |  |
| Information-sharing with school staff and parents | |  |  |  |  |  |  |  |
|  | Yes |  | 125 | (91.2) | 6.7 | (2.2) |  | .063^‡^ |
|  | No |  | 12 | (8.8) | 5.4 | (1.8) |  |  |
| Support for instruction in independent activities | |  |  |  |  |  |  |  |
|  | Yes |  | 53 | (38.7) | 6.6 | (2.3) |  | .753^‡^ |
|  | No |  | 84 | (61.3) | 6.5 | (2.1) |  |  |
| Promoting understanding of CMC among classmates and teachers | |  |  |  |  |  |  |  |
|  | Yes |  | 70 | (51.1) | 6.8 | (2.1) |  | .109^‡^ |
|  | No |  | 67 | (48.9) | 6.2 | (2.3) |  |  |
| Emergency response | |  |  |  |  |  |  |  |
|  | Yes |  | 100 | (73.0) | 6.6 | (2.1) |  | .913^‡^ |
|  | No |  | 37 | (27.0) | 6.5 | (2.5) |  |  |
| Management of medical equipment and supplies | |  |  |  |  |  |  |  |
|  | Yes |  | 90 | (65.7) | 6.5 | (2.3) |  | .729^‡^ |
|  | No |  | 47 | (34.3) | 6.6 | (2.1) |  |  |
| Consultation with parents regarding medical care | |  |  |  |  |  |  |  |
|  | Yes |  | 80 | (58.4) | 6.7 | (2.1) |  | .301^‡^ |
|  | No |  | 57 | (41.6) | 6.3 | (2.3) |  |  |
| Support for the education of students without medical care needs | |  |  |  |  |  |  |  |
|  | Yes |  | 67 | (48.9) | 6.6 | (2.3) |  | .682^‡^ |
|  | No |  | 70 | (51.1) | 6.5 | (2.2) |  |  |
| Providing advice to parents regarding medical care | |  |  |  |  |  |  |  |
|  | Yes |  | 54 | (39.4) | 6.7 | (2.2) |  | .668^‡^ |
|  | No |  | 83 | (60.6) | 6.5 | (2.2) |  |  |
| Development of emergency response manuals | |  |  |  |  |  |  |  |
|  | Yes |  | 53 | (38.7) | 6.5 | (1.9) |  | .692^‡^ |
|  | No |  | 84 | (61.3) | 6.6 | (2.4) |  |  |
| Communication and reporting together with attending physicians, school physicians, and other health care professionals | | | | | | | | |
|  | Yes |  | 45 | (32.8) | 6.8 | (1.8) |  | .351^‡^ |
|  | No |  | 92 | (67.2) | 6.4 | (2.4) |  |  |
| Promoting awareness among school staff | |  |  |  |  |  |  |  |
|  | Yes |  | 31 | (22.6) | 6.6 | (1.9) |  | .925^‡^ |
|  | No |  | 106 | (77.4) | 6.5 | (2.3) |  |  |
| Promoting social development of CMC | |  |  |  |  |  |  |  |
|  | Yes |  | 31 | (22.6) | 6.7 | (2.4) |  | .643^‡^ |
|  | No |  | 106 | (77.4) | 6.5 | (2.2) |  |  |
| Coordination with external agencies (medical, public health, and welfare services) | | | | | | | | |
|  | Yes |  | 18 | (13.1) | 6.4 | (1.6) |  | .833^‡^ |
|  | No |  | 119 | (86.9) | 6.6 | (2.3) |  |  |
| Medical care during the commute to and from school | |  |  |  |  |  |  |  |
|  | Yes |  | 9 | (6.6) | 7.3 | (1.2) |  | .270^‡^ |
|  | No |  | 128 | (93.4) | 6.5 | (2.3) |  |  |
|  |  |  |  |  |  |  |  |  |
| **Collaboration and cooperation** | |  |  |  |  |  |  |  |
| Status of collaboration with teachers | |  |  |  |  |  |  |  |
|  | Collaboration group |  | 114 | (83.2) | 6.9 | (2.0) |  | < .001^‡^ |
|  | Non-collaboration group |  | 23 | (16.8) | 4.8 | (2.5) |  |  |
| Status of collaboration with Yogo teachers | |  |  |  |  |  |  |  |
|  | Collaboration group |  | 90 | (65.7) | 6.9 | (1.9) |  | .034^†^ |
|  | Non-collaboration group |  | 47 | (34.3) | 5.9 | (2.6) |  |  |
| NTCS total score | |  | 137 |  | 70.9 | (10.6) | 0.570 | < .001^§^ |
| Notes: Attachment was recoded into high (always or generally) and low (rarely or not at all). Perceived difficulty in clinical judgment was recoded into difficult (difficult or sometimes difficult) and not difficult (rarely difficult or not difficult). Collaboration with teachers and Yogo teachers was recoded into established (well or generally established) and not established (rarely or not established). † Welch test. ‡ *t*-test. § Pearson’s correlation coefficient. Abbreviations: CMC, children with medical complexity; NTCS, Nurse–Teacher Collaboration Scale; N, number of participants; M, mean; SD, standard deviation. | | | | | | | | |
